# Contributions of symptomatic osteoarthritis and physical function to incident cardiovascular disease

**DOI:** 10.1186/s12891-018-2311-4

**Published:** 2018-11-10

**Authors:** Michela Corsi, Carolina Alvarez, Leigh F. Callahan, Rebecca J. Cleveland, Yvonne M. Golightly, Joanne M. Jordan, Amanda E. Nelson, Jordan Renner, Allen Tsai, Kelli D. Allen

**Affiliations:** 1 College of Medicine, Northeast Ohio Medical University, 4209 OH-44, Rootstown, OH 44272 USA; 2 Thurston Arthritis Research Center, University of North Carolina at Chapel Hill, 3300 Thurston Bldg., CB# 7280, Chapel Hill, NC 27599 USA; 3 Department of Medicine, University of North Carolina at Chapel Hill, 125 MacNider Hall CB# 7005, Chapel Hill, NC 27599 USA; 4 Injury Prevention Research Center, University of North Carolina at Chapel Hill, CB# 7505, 137 E Franklin St, Chapel Hill, NC 27599 USA; 5 Department of Epidemiology, University of North Carolina at Chapel Hill, 170 Rosenau Hall, CB#7400, Chapel Hill, NC 27599 USA; 6 Department of Radiology, University of North Carolina at Chapel Hill, 2006 Old Clinic, CB, Chapel Hill, NC #7510 USA; 7 Center for Health Services Research in Primary Care, Department of Veterans Affairs Healthcare System, 508 Fulton Street, Durham, NC USA

**Keywords:** Osteoarthritis, Function, Cardiovascular disease

## Abstract

**Background:**

Osteoarthritis (OA) is associated with worsening physical function and a high prevalence of comorbid health conditions. In particular, cardiovascular disease (CVD) risk is higher in individuals with OA than the general population. Limitations in physical function may be one pathway to the development of CVD among individuals with OA. This study evaluated associations of symptomatic knee OA (sxKOA), baseline physical function and worsening of function over time with self-reported incident CVD in a community-based cohort.

**Methods:**

Our sample consisted of individuals from the Johnston County Osteoarthritis Project who did not report having CVD at baseline. Variables used to evaluate physical function were the Health Assessment Questionnaire (HAQ), time to complete 5 chair stands, and the 8-ft walk. Worsening function for these variables was defined based on previous literature and cutoffs from our sample. Logistic regression analyses examined associations of sxKOA, baseline function and worsening of function over time with self-reported incident CVD, unadjusted and adjusted for relevant demographic and clinical characteristics.

**Results:**

Among 1709 participants included in these analyses, the mean age was 59.5 ± 9.5 years, 63.6% were women, 15% had sxKOA, and the follow up time was 5.9 ± 1.2 years. About a third of participants reported worsening HAQ score, about two-fifths had worsened chair stand time, half had worsened walking speed during the 8-ft walk, and 16% self-reported incident CVD. In unadjusted analyses, sxKOA, baseline function, and worsening function were all associated with self-reported incident CVD. In multivariable models including all of these variables, sxKOA was not associated with incident CVD, but worsening function was significantly associated with increased CVD risk, for all three functional measures: HAQ odds ratio (OR) = 2.49 (95% confidence interval (CI) 1.90–3.25), chair stands OR = 1.58 (95% CI 1.20–2.08), 8-ft walk OR = 1.53 (95%CI 1.15–2.04). These associations for worsening function remained in models additionally adjusted for demographic and clinical characteristics related to CVD risk.

**Conclusions:**

The association between symptomatic knee osteoarthritis and cardiovascular disease risk was explained by measures of physical function. This highlights the importance of physical activity and other strategies to prevent functional loss among individuals with symptomatic knee osteoarthritis.

## Background

Osteoarthritis (OA) is a key contributor to functional disability that is becoming increasingly prevalent worldwide [1]. Symptomatic knee OA (sxKOA) is associated with functional limitations, which tend to worsen over time [2, 3]. Individuals with OA also have a significantly increased risk of cardiovascular disease (CVD) [4–6]. Individuals with OA tend to have multiple risk factors for CVD, including increased body mass index (BMI), hypertension, physical inactivity, and nonsteroidal anti-inflammatory drug (NSAID) use [5, 7]. For this reason, many hypotheses have been proposed regarding underlying pathophysiological mechanisms connecting OA and CVD, including the role of common molecular or metabolic pathways, chronic low-grade inflammation leading to both conditions, and the development of functional limitations from OA that in turn leads to a lack of physical activity, exacerbating both conditions [7, 8].

Recently, a number of studies have shown an association between physical function and CVD among individuals with OA. Schieir et al. showed that there was a greater risk of CVD in women with arthritis (with participants primarily having OA), compared to women without arthritis; the risk of CVD was further increased in women with both arthritis and physical limitations [9]. Among men in this study, there was only an increased risk of incident CVD for those who reported both arthritis and physical limitations. Together these results suggest that physical function may play a significant role in the development of CVD in patients with arthritis. While this study focused broadly on arthritis, another cohort study found that the relationship between sxKOA and CVD was sustained when controlling for age, obesity, and metabolic factors, yet became insignificant when controlling for functional limitations [10]. However, this was a cross-sectional study, so a causal relationship could not be established. Another cohort study found that individuals with hip or knee OA who used a walking aid due to functional disability had a 30% greater risk of developing CVD than those who did not use a walking aid [11]. Another recent longitudinal cohort study found a dose-response relationship between the number of joints with OA and CVD risk; however, this relationship became non-significant when controlling for difficulty walking [12].

The purpose of this study was to examine associations of sxKOA, baseline physical function and worsening of function over time with self-reported incident CVD in a community-based cohort. In particular, we were interested in understanding whether different measures of physical function explained any relationship between sxKOA and CVD risk. This study adds to the literature in several important ways. First, it is one of few studies to examine the association between OA and CVD risk in a longitudinal analysis. Second, this study has multiple measures of function, including performance-based measures, which to our knowledge have not been used in other longitudinal studies of this topic. This deepens our understanding of how various functional measures may serve as predictors of CVD among individuals with OA. Third, this study examined not only baseline function but also change in function over time; prior studies have not assessed the role of worsening function over time and how this may play in the development of CVD in individuals with OA.

## Methods

### Participants

This study involved participants in the Johnston County Osteoarthritis Project (JoCo OA), an ongoing community-based study focusing on hip and knee OA in a rural population [13]. Participants were civilian, non-institutionalized African-American and Caucasian adults aged 45 years and older selected from six townships within Johnston County, North Carolina. Initial enrollment occurred from 1991 to 1997 (Original Cohort), with first follow-up of this cohort occurring from 1999 to 2003. A second wave of enrollment occurred in 2003–2004 (Enrichment Cohort), aimed at enriching the sample for AA and younger individuals. First follow-up of this group occurred from 2006 to 2011. This research was reviewed and approved by the Institutional Review Board of the University of North Carolina, Chapel Hill; all participants provided written informed consent.

**Fig. 1:**
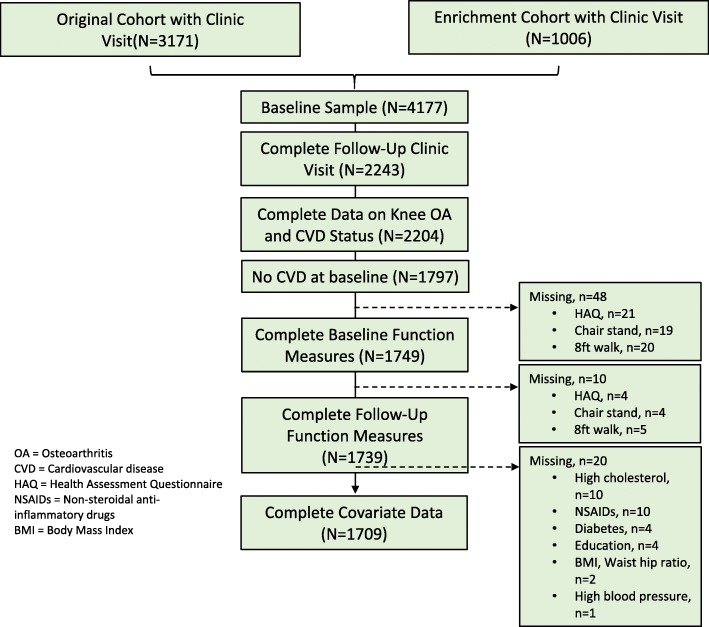
Flow Chart of Participants Included in Analyses

From participants enrolled in the Original Cohort (*N* = 3249) and Enrichment Cohort (*N* = 1141), we excluded individuals who did not have baseline clinic data, follow-up clinic data (due to loss to follow-up), and baseline and follow-up knee OA and CVD status. Then those who self-reported having CVD at baseline were excluded (Fig. 1). Finally, we excluded individuals who were missing baseline or follow-up data for functional tests or covariates, leading to a final sample size of 1709. Complete case analysis (CCA) was used so that only participants with non-missing baseline covariates and physical function status at baseline and follow-up were analyzed. This proportion of participant with missing baseline or follow-up data for analyses was 4.9%, so the impact of bias from their removal is likely to be small and CCA can be conducted regardless of the missing data pattern (Fig. 1) [14].

### Measures

#### Outcome: Incident self-report CVD

Incident CVD was assessed at first follow-up through self-report. The definition of CVD was based on the World Health Organization criteria; due to changes in these criteria, self-report items differed slightly at different time points. For the Original Cohort, CVD at first follow-up was defined as having a heart attack, stroke, circulation or other heart problem. For the Enrichment Cohort, the incident CVD definition at first follow-up was expanded to include angina and congestive heart failure.

#### Symptomatic knee OA

JoCo OA participants had anteroposterior (Original Cohort enrollment) or posteroanterior (Enrhichment Cohort enrollment) radiographs taken of both knees while weight-bearing using a Synaflexer® positioning device. All radiographs were read for Kellgren-Lawrence (K-L) score by a single bone and joint radiologist (JBR) without regard to participant’s clinical status. Intrarater reliability and interrater reliability, assessed with another trained radiologist, were both high (weighted kappas were 0.89 and 0.86, respectively). For the purpose of this study, radiographic KOA was defined as a K-L grade ≥ 2. To assess joint symptoms, participants were asked: “On most days, do you have pain, aching, or stiffness in your…right/left knee.” Participants responding “yes” to this question for a joint with radiographic OA were considered to have sxKOA.

#### Function measures

We included three measures of physical function: chair stands, 8-ft walk, and the Health Assessment Questionnaire Disability Index (HAQ).

##### Health assessment questionnaire

The HAQ is a measure of self-reported disability, assessing ability to perform typical eight daily tasks (dressing, arising, eating, walking, reaching, gripping, chores, and hygiene) during the past 7 days [15]. Answers for each question are scored from 0 to 3, with 0 being no disability and 3 being complete disability. Per scoring guidelines, mean HAQ score was calculated for each participant if six or more of the eight categories were non-missing. We categorized baseline mean HAQ as follows: HAQ = 0, 0 < HAQ < 1, or HAQ ≥ 1, based on definitions previously used with JoCo OA data [16]. Based on the previously established minimum clinically important difference in HAQ score [15, 17, 18], we defined a clinically significant change as baseline +/− 0.22; individuals whose baseline scores rose by 0.22 or more were classified as worsening, and those whose baseline scores fell by 0.22 or more were classified as improving. If change in speed did not meet these values, participants were classified as having stayed the same. The groups were then dichotomized into improved/stayed the same, or worsened.

##### Chair stands

Based on previously established protocols [19, 20], we assessed time for participants to complete 5 chair stands. Participants were seated in a chair with feet touching the floor and asked to rise without the use of arms as support. To ensure this, participants were asked to cross their arms at their wrists and hold them tight to their chests throughout the test. Participants who were unable to rise from a chair by themselves or scoot forward or stand up without using their arms were classified being unable to complete this test. For those able to perform this task, time taken to complete the 5 chair stands was recorded in seconds. We then categorized times into quartiles, based on baseline scores of those without sxKOA in our study sample. These thresholds were also determined separately for males and females due to evidence of differences in performance between these two groups [21]. For males, these cutoffs were time < 8.4 s (quartile 1, Q1), 8.4 s < time < 10.2 s (quartile 2, Q2), 10.2 s < time < 12.9 s (quartile 3, Q3), 12.9 s < time (quartile 4, Q4), or unable to complete all five chair stands. For females, these cutoffs were time < 9.0 s (Q1), 9.0 s < time < 11.3 s (Q2), 11.3 s < time < 14.1 s (Q3), 14.1 s < time (Q4), or unable to complete all five chair stands. We then categorized participants as either having worsened (moved up a quartile from baseline) or stayed the same / improved (remained in the same quartile or moved down a quartile from baseline) at the time of first follow-up. Participants unable to complete the chair stands at baseline were categorized as follows: staying the same / improving if they became able to complete at follow-up and worsening if they remained unable to complete at follow-up.

##### 8-foot walk

Using previously established procedures [20, 22], participants were asked to perform two trials in which they walked the 8-ft at their normal pace; times of the two trials were averaged and converted to gait speed (m/s) and kept continuous for the baseline measure. We categorized participants as either worsening or staying the same/improving in gait speed at follow-up. We defined worsening as a decrease of 0.1 m/s based on previous literature suggesting this may be a clinically relevant decline [23, 24].

##### Covariates

Variables that could potentially confound the associations between sxKOA, function and CVD were included in multivariable models. These included: baseline enrollment group (Original vs. Enrichment cohort), age, gender, race (African American vs. Caucasian), education (< high school vs. ≥ high school), body mass index (BMI), waist to hip ratio (WHR), self-reported presence of diabetes, hypertension, or high cholesterol, and self-reported nonsteroidal anti-inflammatory drugs (NSAIDs) use at baseline. BMI, diabetes mellitus, hypertension and high cholesterol are often considered aspects of metabolic syndrome. These variables were included in our model as previous research has indicated there may be underlying metabolic contributions to the development of comorbid CVD in individuals with OA [8]. NSAID use was also chosen as a possible confounder as it too has been indicated as a possible contributor to increased CVD risk in individuals with OA [7].

### Statistical analysis

Descriptive statistics were calculated for all participants in the final analytic sample. Logistic regression models were used to model the odds of incident CVD. These population-averaged models for non-normal (binomial) measures were fit to each of the three physical function measure analyses. First, unadjusted odds ratios (OR) and 95% confidence intervals (CI) were computed to examine associations of sxKOA, baseline function, and worsening function with incident CVD. Second, multivariable models that jointly examined associations of sxKOA, baseline function and worsening function with incident CVD (Model 1) were conducted. Third, multivariable models that included Model 1 variables along with relevant demographic and health covariates (Model 2) were conducted. Models 1 and 2 were assessed for the interaction of sxKOA with physical function (both the baseline measure and the follow-up worsening indicator) at the 0.05 significance level. All statistical computations were performed using SAS, version 9.4 (Cary, NC).

## Results

Participant characteristics are summarized in Table 1. Almost a quarter of our sample consisted of the Enrichment cohort, and overall mean time to follow-up was 5.9 ± 1.2 years. The sample comprised 63.6% women, 27.8% African Americans, and 25.9% with less than a high school education, while mean age of participants was 59.5 ± 9.5 years. The mean BMI was 29.3 ± 6.0 kg/m^2^, with over three quarters of participants being overweight (38.2%) or obese (38.4%). At baseline, approximately a tenth of participants had diabetes, a third had hypertension, a fifth had high blood cholesterol, and almost a third reported NSAID use. Baseline sxKOA was present for approximately 15% of participants, and approximately 16% of participants developed CVD by their first follow-up. Individuals with sxKOA at baseline had 1.50 times the unadjusted odds of incident CVD (95% CI 1.08–2.08).

**Table 1:** Descriptive Characteristics of Study Sample (*N* = 1709)

Characteristic	Mean (SD) or %
Baseline	
% Original cohort	75.6
Demographic	
Mean (SD) Age	59.5 (9.5)
% Women	63.6
% African American	27.8
% with < 12 Years Education	25.9
Health Related	
Mean (SD) Body Mass Index (kg/m^2^)	29.3 (6.0)
Mean (SD) Waist to Hip Ratio	0.87 (0.09)
% Hypertension	32.9
% Diabetes	8.7
% High cholesterol	20.8
OA Related	
% Non-steroidal Anti-inflammatory Drug Use	31.5
% Symptomatic Osteoarthritis	15.4
Function Related	
Mean Health Assessment Questionnaire; HAQ (SD)	0.27 (0.47)
% 0 = HAQ	58.6
% 0 < HAQ < 1	30.8
% 1 ≤ HAQ	10.6
Mean (SD) Speed During 8 ft. Walk (m/s)	0.87 (0.26)
Mean (SD) Chair Stand Times (s)	12.2 (4.8)
% Q1^a^	20.5
% Q2	23.1
% Q3	23.5
% Q4	27.5
% Unable to Complete	5.4
Follow-up	
Mean (SD) years to follow-up	5.9 (1.2)
% Worsened HAQ	33.0
% Worsened 8 ft. Walk speed	49.6
% Worsened Chair Stand Time	42.2
% Incident Cardiovascular Disease	15.9

^a^5 Chair stand time quartlies defined from the non-exposure subsample without sxKOA at baseline: Males: Q1 (time < =8.4 s), Q2 (8.4 s < time < =10.2 s), Q3 (10.2 s < time < =12.9 s), Q4 (12.9 s < time)"; Females: Q1 (time < =9.0 s), Q2 (9.0 s < time < =11.3 s), Q3 (11.3 s < time < =14.1 s), Q4 (14.1 s < time)

*SD* standard deviation, *OA* Osteoarthritis

### HAQ

Regarding mean HAQ scores, over half of participants (58.6%) had a score of zero, almost a third (30.8%) had 0 < HAQ < 1, and about a tenth (10.6%) had 1 ≤ HAQ at baseline. About a third (33.0%) of participants worsened by 0.22 units or more in mean HAQ score by first follow-up (which corresponds to the minimum clinically important difference), with the remainder staying the same or improving.

There were no significant interactions of sxKOA with baseline or worsening HAQ scores, so overall main effects are reported (Table 2). In unadjusted analyses, participants with baseline HAQ scores > 0 had 30–90% higher odds of incident CVD compared to those with HAQ = 0; those with worsening HAQ scores over time had 2.5 times greater odds of incident CVD. In multivariable Model 1, baseline HAQ score ≥ 1 and worsening HAQ continued to have significantly greater odds of incident CVD, but sxKOA was no longer significantly associated with CVD risk in this model. These associations were similar in the fully adjusted model (Model 2), in which age and self-reported diabetes were also associated with incident CVD.

**Table 2:** Unadjusted and Adjusted Associations of sxKOA and HAQ Scores with Incident CVD

Variable	Unadjusted	Model 1^a^	Model 2^a^
	OR (95% CI)	OR (95% CI)	OR (95% CI)
sxKOA vs. no sxKOA at baseline	**1.50 (1.08, 2.08)**	1.14 (0.80, 1.61)	1.10 (0.76, 1.61)
Baseline 0 < HAQ < 1 vs. HAQ = 0	1.29 (0.97, 1.72)	1.17 (0.87, 1.57)	1.08 (0.79, 1.47)
Baseline 1 ≤ HAQ vs. HAQ = 0	**1.94 (1.32, 2.85)**	**1.91 (1.27, 2.86)**	**1.82 (1.18, 2.79)**
Worsened HAQ vs. unchanged or improved	**2.51 (1.93, 3.27)**	**2.49 (1.90, 3.25)**	**2.35 (1.79, 3.10)**
Age: 1 year increase			**1.02 (1.00, 1.03)**
Gender: Female vs. Male			1.28 (0.91, 1.80)
Race: Black vs. White			1.13 (0.83, 1.54)
Education: <HS vs. HS or greater			0.89 (0.65, 1.24)
Cohort: Enrichment vs. Original			0.96 (0.69, 1.33)
BMI: 1 kg/m^2^ increase			0.98 (0.95, 1.00)
WHR: 0.1 unit increase			1.15 (0.98, 1.36)
Diabetes vs. not			**1.84 (1.21, 2.80)**
Hypertension vs. not			1.10 (0.81, 1.48)
High cholesterol vs. not			1.03 (0.74, 1.44)
NSAIDs vs. not			1.27 (0.94, 1.71)

*CI* confidence interval, *sxKOA* symptomatic knee osteoarthritis, *HAQ* health assessment questionnaire, *HS* high school, *BMI* body mass index, *WHR* waist-to-hip ratio, *NSAIDs* non-steroidal anti-inflammatory drugs

^a^Models 1 and 2 are adjusted for all variables with data listed in the column

### Chair stands

For chair stands, 5.4% of participants were unable to complete the test at baseline, and the quartile cutoff distribution (fastest to slowest times) was 20.5%, 23.1%, 23.5 and 27.5%. About 42% of participants worsened by moving up a quartile (or becoming unable) in chair stand performance at follow-up, with the remaining staying the same or improving.

There were no significant interactions of sxKOA with baseline or worsening chair stand time and so overall main effects are reported (Table 3). Compared to participants in the lowest quartile (Q1) of chair stand time, those in higher quartiles (Q2, Q3, and Q4) and those unable to complete the test had significantly elevated unadjusted odds of incident CVD; worsening chair stand performance over time was also associated with increased incident CVD. In multivariable Model 1, similar associations with incident CVD remained for baseline chair stand quartiles and worsening chair stand time, but sxKOA was no longer significantly associated with incident CVD risk in this model. These associations were similar in the fully adjusted model (Model 2), although inability to complete chair stands was no longer significantly associated with incident CVD risk; sex and diabetes were also associated with increased CVD risk.

**Table 3:** Unadjusted and Adjusted Associations of sxKOA and Chair Stands with Incident CVD

Variable	Unadjusted	Model 1^a^	Model 2^a^
	OR (95% CI)	OR (95% CI)	OR (95% CI)
sxKOA vs. no sxKOA at baseline	**1.50 (1.08, 2.08)**	1.28 (0.90, 1.80)	1.24 (0.86, 1.79)
Baseline 5 chair stand time Q2 vs. Q1**	**1.77 (1.14, 2.75)**	**1.82 (1.17, 2.83)**	**1.77 (1.13, 2.78)**
Baseline 5 chair stand time Q3 vs. Q1**	**1.93 (1.25, 2.98)**	**2.04 (1.31, 3.16)**	**1.79 (1.14, 2.81)**
Baseline 5 chair stand time Q4 vs. Q1**	**1.85 (1.21, 2.82)**	**2.02 (1.30, 3.15)**	**1.65 (1.02, 2.65)**
Baseline 5 chair stand time unable vs. Q1**	**2.50 (1.36, 4.58)**	**2.14 (1.16, 3.98)**	1.71 (0.89, 3.27)
Worsened 5 Chair stand time vs. unchanged or improved	**1.50 (1.15, 1.94)**	**1.58 (1.20, 2.08)**	**1.45 (1.09, 1.93)**
Age: 1 year increase			1.01 (1.00, 1.03)
Gender: Female vs. Male			**1.46 (1.05, 2.04)**
Race: Black vs. White			1.11 (0.82, 1.52)
Education: <HS vs. HS or greater			0.95 (0.68, 1.31)
Cohort: Enrichment vs. Original			1.06 (0.77, 1.47)
BMI: 1 kg/m^2^ increase			0.98 (0.96, 1.01)
WHR: 0.1 unit increase			1.16 (0.99, 1.36)
Diabetes vs. not			**1.91 (1.26, 2.89)**
Hypertension vs. not			1.13 (0.84, 1.52)
High cholesterol vs. not			1.03 (0.74, 1.44)
NSAIDs vs. not			1.30 (0.97, 1.74)

*CI* confidence interval, *Q* quarter, *sxKOA* symptomatic knee osteoarthritis, *HS* high school, *BMI* body mass index, *WHR* waist-to-hip ratio, *NSAIDs* non-steroidal anti-inflammatory drugs

^a^Models 1 and 2 are adjusted for all variables with data listed in the column

### 8-foot walk

The mean baseline gait speed during the 8-ft walk was 0.87 m/s (SD = 0.26). About half (49.6%) of participants worsened by decreasing 0.1 m/s or more in mean gait speed by first follow-up. The measure of baseline gait speed showed a significant interaction with baseline sxKOA status, so effects of baseline gait speed are shown separately for participants without and with sxKOA (Table 4). Results for baseline gait speed are presented in units of 0.3 m/s difference; this was selected because it approximated 1 standard deviation for the distribution of baseline gait speeds. For participants without sxKOA, unadjusted odds of incident CVD were higher for those with a slower gait speed at baseline. In contrast, for participants with sxKOA, baseline gait speed was not associated with incident CVD. In unadjusted analyses, there was no association between worsening gait speed and incident CVD.

**Table 4:** Unadjusted and Adjusted Associations of sxKOA and 8-Foot Walk with Incident CVD

Variable	Unadjusted	Model 1^a^	Model 2^a^
	OR (95% CI)	OR (95% CI)	OR (95% CI)
Baseline 0.3 m/s (1SD) decrease in mean 8 ft. walk speed among those without sxKOA	**1.46 (1.23, 1.74)**	**1.64 (1.35, 1.99)**	**1.46 (1.17, 1.82)**
Baseline 0.3 m/s (1SD) decrease in mean 8 ft. walk speed among those with sxKOA	0.91 (0.62, 1.33)	0.99 (0.67, 1.47)	0.90 (0.59, 1.36)
Worsened 8 ft. walk vs. unchanged or improved	1.12 (0.87, 1.45)	**1.53 (1.15, 2.04)**	**1.47 (1.07, 2.01)**
Age: 1 year increase			1.01 (0.99, 1.03)
Gender: Female vs. Male			1.35 (0.96, 1.91)
Race: Black vs. White			1.05 (0.77, 1.44)
Education: <HS vs. HS or greater			0.92 (0.66, 1.27)
Cohort: Enrichment vs. Original			1.10 (0.79, 1.54)
BMI: 1 kg/m^2^ increase			0.98 (0.96, 1.01)
WHR: 0.1 unit increase			1.15 (0.98, 1.36)
Diabetes vs. not			**1.80 (1.19, 2.71)**
Hypertension vs. not			1.11 (0.83, 1.49)
High cholesterol vs. not			1.09 (0.79, 1.52)
NSAIDs vs. not			**1.34 (1.00, 1.80)**

*CI* confidence interval, *Q* quarter, *sxKOA* symptomatic knee osteoarthritis, *HS* high school, *BMI* body mass index, *WHR* waist-to-hip ratio, *NSAIDs* non-steroidal anti-inflammatory drugs

^a^Models 1 and 2 are adjusted for all variables with data listed in the column

In multivariable Model 1, slower baseline gait speed remained associated with incident CVD only for those without sxKOA. Worsening gait speed was also associated with incident CVD in Model 1. These associations were similar in the fully adjusted Model 2, in which NSAID use and self-reported diabetes were also associated with incident CVD.

## Discussion

In this analysis, we examined the unique contributions of sxKOA, baseline function and worsening of function with incident CVD risk. With respect to sxKOA, we found significant associations with incident CVD risk in unadjusted analyses, but not in multivariable analyses adjusting for functional variables. There were significant associations of functional variables with incident CVD risk, and in particular, worsening of function variables over time was consistently associated with CVD risk in multivariable models. The association of one function variable, 8-ft walk, differed between those with and without sxKOA, being important only in the latter group.

The finding of a significant bivariate association of sxKOA with CVD risk is in agreement with results of prior studies [5, 10–12, 25]. Importantly, the longitudinal nature of this association supports that sxKOA predicts development of future CVD. In multivariable analyses including function variables, even prior to adjustment for other confounders, sxKOA was no longer significantly associated with CVD risk. This confirms another recent longitudinal study in which the association of number of joints with OA with CVD risk was explained by self-reported difficulty walking at baseline [12]. These studies indicate that function is at least one key contributor to CVD risk among individuals with sxOA. Knowledge of this underlying mechanism is extremely important, as it points to a potential intervention approach. In particular, physical activity programs can significantly improve functional outcomes among individuals with sxKOA [26] and therefore may confer an important benefit regarding downstream CVD risk, particularly since physical activity also improves a number of metabolic factors leading to CVD.

There were a number of interesting findings regarding associations of functional measures with CVD risk. First, it is notable that all of the function measures (with the exception of 8-ft walk for people with sxKOA) were associated with increased CVD risk, suggesting that both performance-based measures and self-report measures (e.g., HAQ) may be helpful markers of CVD risk; this adds to prior studies that have shown positive associations with self-report function measures. Second, even when baseline function and change in function were included in the same model, change in function consistently remained significantly associated with incident CVD risk. This also adds to prior studies, which have focused on functional status at a single time point. These results illustrate the importance of functional decline in the prediction of CVD, regardless of baseline functional status, and further illustrates the importance of physical activity programs that can slow the progression of functional decline. Third, functional measures (particularly change in function over time) continued to have significant associations with CVD risk even in fully adjusted models that included a number of factors related to metabolic syndrome (e.g, BMI, WHR, diabetes). This is important since metabolic syndrome has been another proposed mechanism underlying the association between OA and CVD [7, 8]. Kendzerska et al. also found that self-reported walking-related disability continued to predict CVD risk even when adjusting for BMI and other metabolic factors [12]. This evidence supports that function plays a unique role in the relationships between sxKOA and CVD risk. Fourth, there was an interaction between sxKOA and the 8-ft walk variable: those without sxKOA who had worse baseline 8-ft walk speed were at a greater risk of developing incident CVD, but for those with sxKOA, baseline 8-ft walk performance was not a predictor of incident CVD risk. We hypothesize this result may be due to the fact that our sxKOA group had worse baseline walking speeds than the non-OA group at baseline (.75 m/s vs. 89 m/s), which confirms prior studies [27, 28]. Since walking speed was low overall among those with sxKOA, the variability within that lower range may not have been associated with differential CVD risk.

The association of between baseline chair stand time categories and increased CVD risk was non-linear. Relative to participants in the best baseline chair stand category (Q1), odds of CVD risk were elevated more for middle categories (Q2, Q3) than for those in the worst categories (Q4, unable to complete). However, the odds ratios were still relatively similar (1.65–1.79 in fully adjusted models). The lack of completely linear relationship may be due to the relatively small number of participants in each baseline chair stand group.

Strengths of this study include a community-based cohort including African American and Caucasian men and women, the use of both self-reported and performance-based measures of physical function, the longitudinal approach, and the inclusion of worsening variables for physical function measures. However, there are several limitations to note. First, CVD and function measures at follow-up were assessed at a single time point; therefore, we could not ascertain the specific time of occurrence, and it is possible that functional decline occurred after a CVD diagnosis or event within the follow-up period. Second, incident CVD was assessed via self-report. Although this method allows data for large samples to be obtained readily and cost-effectively, this can result in less accurate representation of true CVD. Strategies were employed in data collection in order to maximize self-report accuracy of CVD. These included impartial and standardized phrasing of the National Health Interview Survey question, ensuring that respondents understood the question completely, and that adequate amount of time for recall was given. However, there may still be instances of inaccurate reporting (either over-reporting or under-reporting). Third, the incident CVD variable definition differed somewhat between cohorts, with angina and congestive heart only being included for the Enrichment Cohort. Fourth, some data were missing due to loss-to-follow-up, and it is possible that individuals with missing follow-up data were less healthy and had more functional limitations than those with complete data.

## Conclusion

Overall, our study indicates that function is a key contributor to the association between symptomatic knee osteoarthritis and cardiovascular disease risk. Further, worsening of function over time seems to have a particularly important role. Physical activity and structured exercise programs can substantially improve function and are already key recommendations for OA management [26]. Unfortunately, many people with symptomatic knee osteoarthritis remain physically inactive [29], placing them at risk for functional loss, and perhaps subsequent elevation of cardiovascular disease risk as a result. These results further elevate the importance of efforts to enhance physical activity among individuals with symptomatic knee osteoarthritis and also highlight the importance of regular physical function assessment.

## Abbreviations

BMI: Body Mass Index
CCA: Complete case analysis
CI: Confidence interval
CVD: Cardiovascular disease
HAQ: Health Assessment Questionnaire
JoCo OA: Johnston County Osteoarthritis Project
K-L: Kellgren Lawrence
NSAID: Non-steroidal Anti-Inflammatory Drug
OA: Osteoarthritis
OR: Odds ratio
Q: Quartile
SD: Standard deviation
SxKOA: Symptomatic knee osteoarthritis
WHR: Waist to Hip Ratio
